# Supplementary material for: DNA Double-Strand Break Repair Inhibitors: YU238259, A12B4C3 and DDRI-18 Overcome the Cisplatin Resistance in Human Ovarian Cancer Cells, but Not under Hypoxia Conditions
Source: Curr Issues Mol Biol. 2023 Sep 28;45(10):7915–32. doi: 10.3390/cimb45100500 (PMC10605129; doi:10.3390/cimb45100500)
Supplement: Supplementary file 1 [file cimb-45-00500-s001.zip › cimb-2639102-supplementary.pdf]

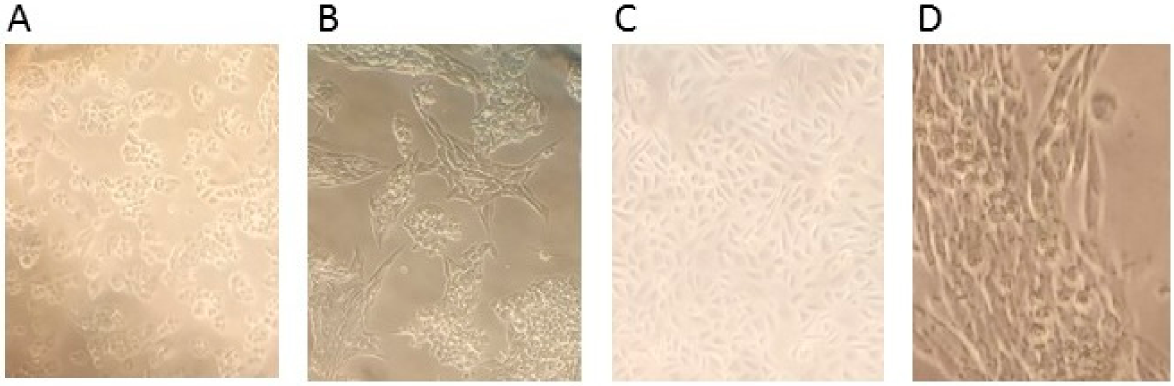

Figure S1. Microscopic images of ovarian cancer cell lines: A2780 (A), A2780cis (B), PEA1 (C) and PEA2 (D).

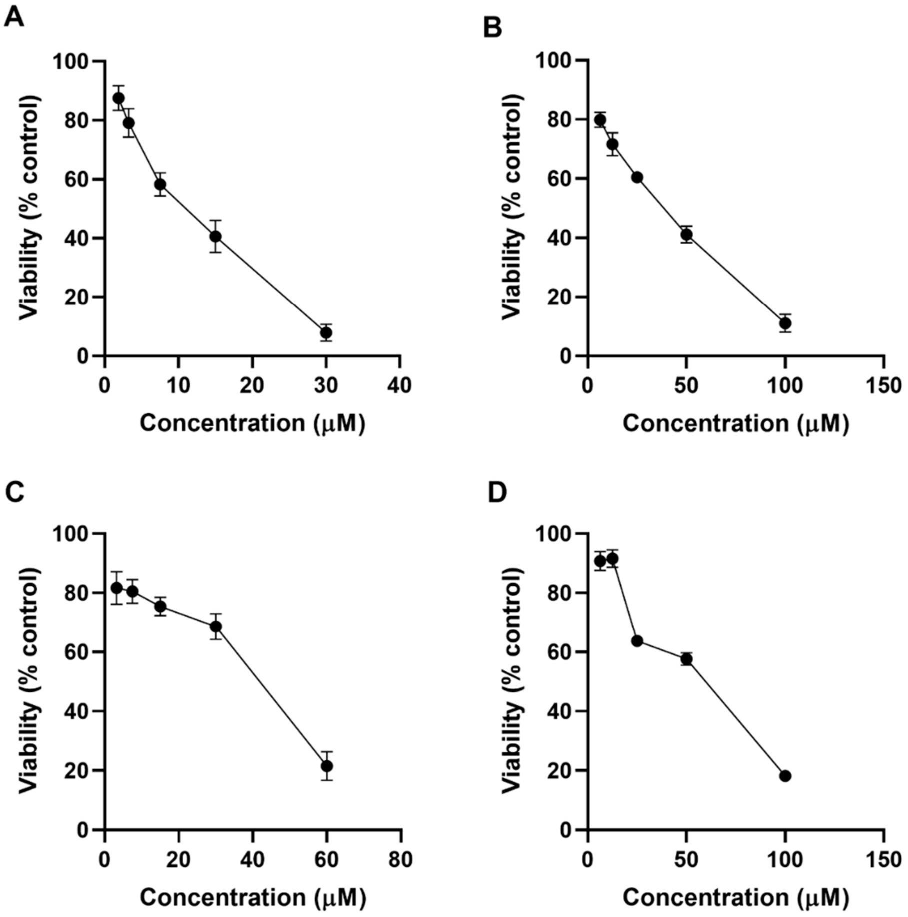

Figure S2. DNA double-strand break inhibitors (DRIs) increase the cytotoxic effect of CDDP (A, C) and VP-16 (B, D) on A2780 (A, B) and A2780cis (C, D) cell lines. Treatment with each of compounds caused concentration-dependent growth inhibition on both of cell lines. Cells were stained with Cell Counting Kit-8 and the OD_{450nm} was determined. Results are means ± SEM of at least 3 experiments.

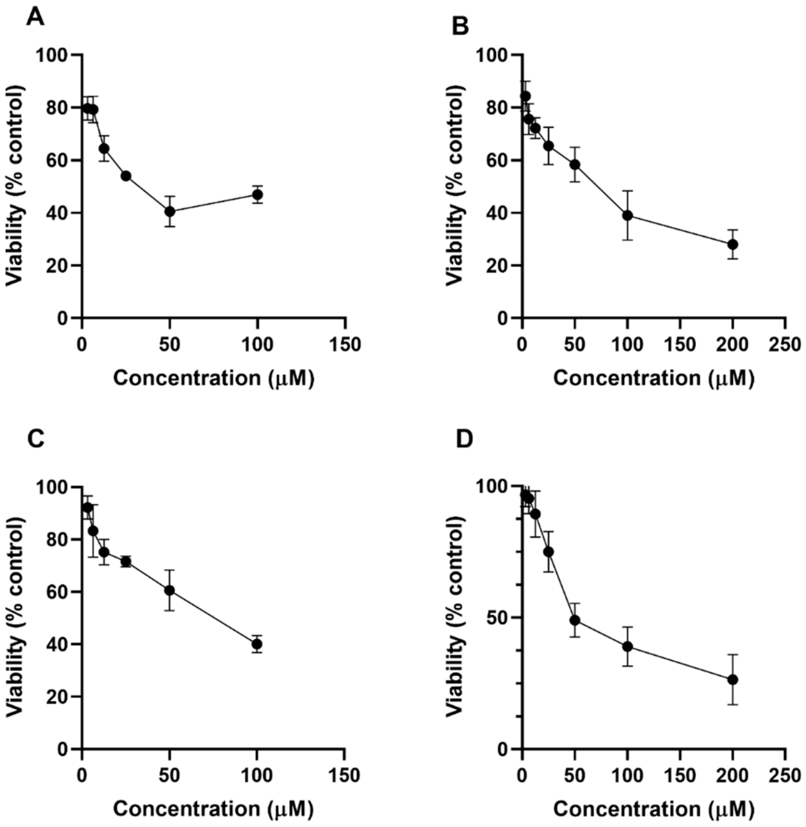

Figure S3. DNA double-strand break inhibitors (DRIs) increase the cytotoxic effect of CDDP (A, C) and VP-16 (B, D) on PEA1 (A, B) and PEA2 (C, D) cell lines. Treatment with each of compounds caused concentration-dependent growth inhibition on both of cell lines. Cells were stained with Cell Counting Kit-8 and the OD_{450nm} was determined. Results are means ± SEM of at least 3 experiments.

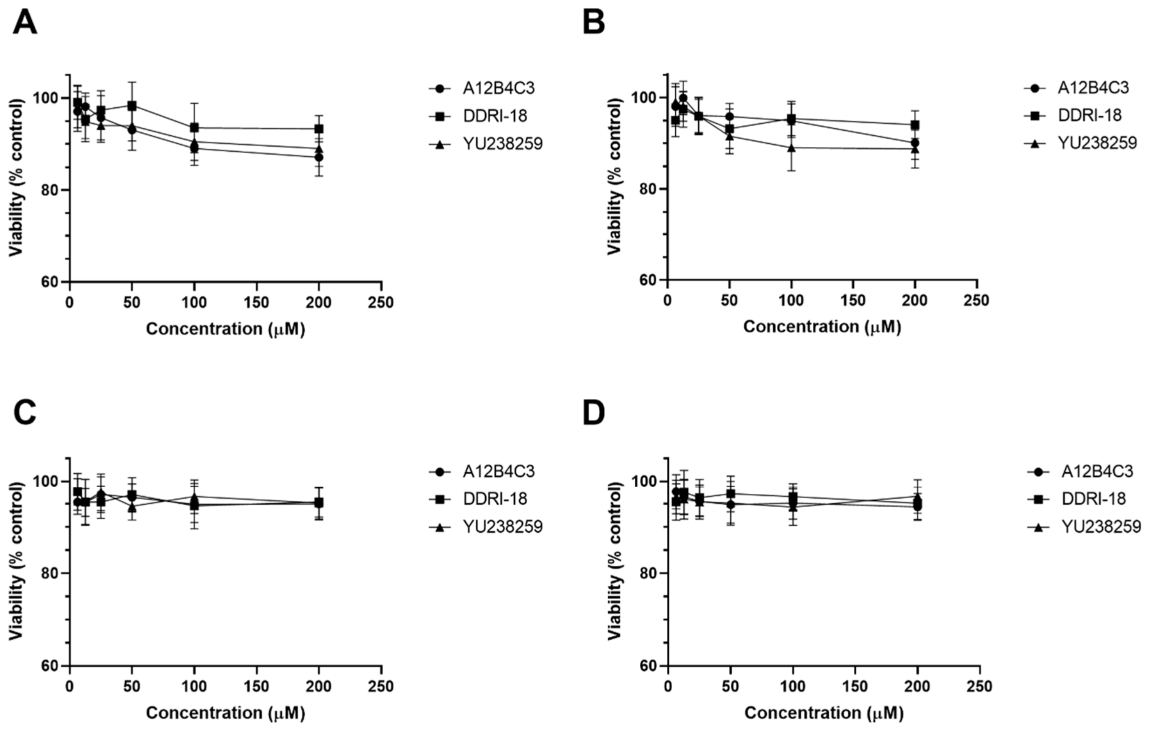

Figure S4. Double-strand break inhibitors (DRIs) : YU238259, DDRI-18 and A12B4C3 does not decrease the viability of ovarian cancer cells. Cells were treated with 10 μM YU238259 (filled triangle, solid line), DDRI-18 (filled square, solid line) or 5 μM A12B4C3 (filled circle, solid line). Cells were then stained with Cell Counting Kit-8 and the OD_{450nm} was determined. Results are means ± SEM of at least 3 experiments. Results are presented for: A – A2780, B – A2780cis, C – PEA1, D – PEA2.

Experimental scheme 1 (A2780 and A2780cis cell lines)

The IC_{50} values determined for the CDDP/VP-16 combination were compared with the values obtained after the introduction of HR or NHEJ inhibitors. After introducing DRIs to the scheme of treatment, the viability of cells decreased compared to samples treated only with CDDP/VP-16 (Tab. S1). The Rf parameter was determined by comparing the IC_{50} values obtained for the variants without the inhibitor and with the inhibitor. In most cases, we obtained Rf >1. Slightly higher sensitization rates were obtained for the resistant ES1 variant compared with the sensitive variant. Although we did not observe a significant effect of sensitization with YU238259 on A2780 cells (Rf = 0.94), results obtained for CDDP-resistant cells was promising (Rf = 2.52). A12B4C3 caused sensitization in both cell lines: 1.2 and 1.62 fold for A2780 and A2780cis cells, respectively. Treatment with DDRI-18 resulted in Rf = 1.60 for A2780 cells and Rf = 1.99 for its counterpart, A2780cis (Tab. S1).These results indicate the role of inhibitors in sensitizing ES1 line cells to the CDDP/VP-16 combination.

Based on the analysis of survival curves, the CI parameter indicating the type of interactions between the drugs was determined. The synergism of CDDP/VP-16 effects increases with increasing

concentrations of these drugs for the A2780 line. No such relationship was observed for the A2780cis line. The introduction of NHEJ inhibitors caused a decrease in the CI parameter, i.e. a change in the interaction between the drugs towards synergism. HR inhibitor YU238259, also had a similar effect (Tab. S2).

Table S1. Comparison of IC_{50} and Rf values obtained for A2780 and A2780cis cells after incubation with a combination of CDDP/VP-16 and DRIs. Rf >1-sensitization.

		A2780		A2780cis	
		IC_{50} (μM)	Rf	IC_{50} (μM)	Rf
Drugs	CDDP/VP-16	16.16	-	35.85	-
Drugs + NHEJ inhibitor	CDDP/VP-16/ YU238259	17.19	0.94	14.23	2.52
Drugs + HR inhibitor	CDDP/VP-16/ A12B4C3	13.39	1.20	22.06	1.62
	CDDP/VP-16/ DDRI-18	10.12	1.60	18.05	1.99

Table S2. CI values obtained for A2780 and A2780cis cells after incubation with CDDP/VP-16 and DRIs. CI values <1 and CI >1 indicate synergism and antagonism, respectively. Indications: ↓ - decrease in CI values, ↑ - increase in CI values.

Drugs concentration CDDP/VP-16 (μM)	CI (for drugs)	CI Drugs + YU238259	CI Drugs + A12B4C3	CI Drugs + DDRI-18
		A2780		
9.49 (¼+¼ IC_{50})	5.86	↓2.50	↓3.09	↓3.98
19 (½+½ IC_{50})	0.83	↓0.57	↑0.92	↓0.73
38 (1+1 IC_{50})	1.15	↓0.56	↓0.54	↓0.68
76 (2+2 IC_{50})	0.61	↓0.38	↓0.50	↓0.40
152 (4+4 IC_{50})	0.52	↑0.65	↑1.00	↓0.42
		A2780cis		
11.3 (¼+¼ IC_{50})	0.53	↓0.44	↓0.39	↓0.48
22.5 (½+½ IC_{50})	0.83	↓0.61	↓0.79	↓0.61
45 (1+1 IC_{50})	0.66	↓0.49	↓0.65	↓0.49
90 (2+2 IC_{50})	0.86	↓0.66	↓0.71	↓0.85
180 (4+4 IC_{50})	1.55	↓1.22	↓1.15	↓0.94

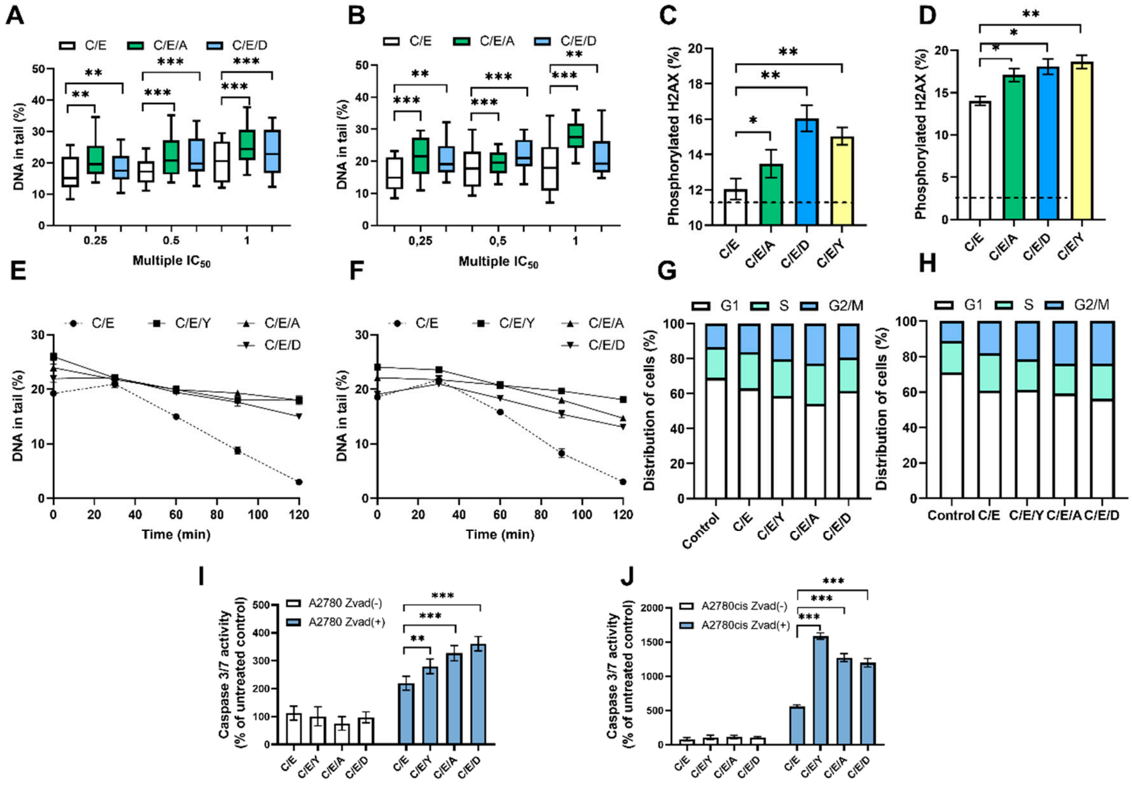

Figure S5. In normoxia conditions, DRIs are able to improve the activity of CDDP/VP-16 combination against human ovarian cancer cells A2780 (A, C, E, G, I) and A2780cis (B, D, F, H, J). DRIs significantly increase CDDP/VP-16 induced level of DNA damage (A, B), the level of phosphorylated H2AX (C, D), modulate DNA repair (E, F), affect the distribution of cell cycle (G, H) and caspases 3 and 7 activity (I, J) . Results are presented as the mean ±SEM (A – B) or mean ±SD (C – J) of 3 independent experiments, * - $p<0.05$; **-$p<0.01$; ***-$p<0.001$.

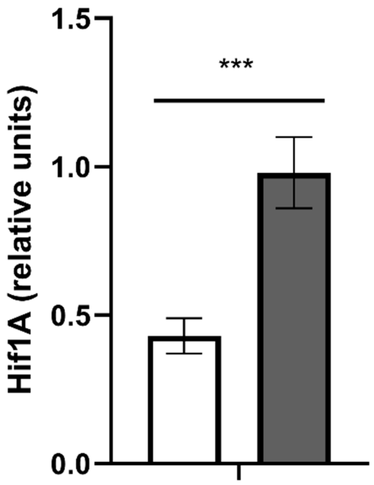

Figure S6. The level of transcription factor Hif1A is elevated under hypoxia conditions. ES 2 cells were cultured under aerobic (white bar) and anaerobic (gray bar) conditions for a minimum of 48 hours. Hif1A levels were determined using a commercial Hif1A Human ELISA Kit. Results are presented as the mean ±SD of 3 independent experiments, * - p<0.05; **-p<0.01; ***-p<0.001.

Table S3. Expression levels of genes involved in DDR in PEA1 cells under hypoxia conditions. Comparison to control - expression levels under aerobic conditions.

Decreased gene expression			Increased gene expression		
Decrease ≥2x			Increase ≥2x		
**Gene**	**Expression**	**Pathway**	**Gene**	**Expression**	**Pathway**
BRCA1	-2.25	ATM/ATR pathway	GADD45A	3.13	DNA repair
CDKN1A	-2.31	Apoptosis	HUS1	2.57	ATM/ATR pathway
CRY1	-2.35	DNA repair	RAD21	2.26	DNA repair
MLH1	-3.80	MMR	**Increase 1,5 - 2**		
PPP1R15A	-2.70	Apoptosis	**Gene**	**Expression**	**Pathway**
TP73	-2.15	MMR	MLH3	1.71	MMR
XPC	-3.73	NER	SUMO1	1.55	DNA repair
XRCC3	-11.63	DNA repair	UNG	1.68	BER
**Decrease 1,5 - 2x**					
**Gene**	**Expression**	**Pathway**			
ABL1	-1.59	MMR			
BAX	-1.70	Apoptosis			
CDC25C	-1.68	Cell cycle			
CHEK1	-1.66	ATM/ATR pathway			

FANCA	-1.67	DNA repair
GADD45G	-1.60	DNA repair
NBN	-1.89	DSBs repair
RAD1	-1.87	DNA repair
RBBP8	-1.60	DNA repair
TP53	-1.53	ATM/ATR pathway
XPA	-1.77	NER

Table S4. Expression levels of genes involved in DDR in PEA2 cells under hypoxia conditions. Comparison to control - expression levels under aerobic conditions.

Decreased gene expression			Increased gene expression		
Decrease ≥5x			Increase ≥5x		
**Gene**	**Expression**	**Pathway**	**Gene**	**Expression**	**Pathway**
BARD1	-8.08	ATM/ATR pathway	ERCC1	12.96	NER
BLM	-8.15	DSBs repair	PPP1R15A	8.11	Apoptosis
BRCA1	-8.55	ATM/ATR pathway	XRCC3	5.25	DNA repair
BRIP1	-9.50	DNA repair	**Increase 2 - 5x**		
CDC25A	-6.00	ATM/ATR pathway	BBC3	4.34	ATM/ATR pathway
CDC25C	-11.67	Cell cycle	DDIT3	4.33	ATM/ATR pathway
CHEK1	-7.15	ATM/ATR pathway	GADD45G	4.78	DNA repair
CHEK2	-6.65	ATM/ATR pathway	XPC	3.55	NER
EXO1	-22.35	MMR			
FANCD2	-20.05	ATM/ATR pathway			
FANCG	-5.69	DSBs repair			
FEN1	-9.91	BER			
H2AFX	-5.75	ATM/ATR pathway			
PCNA	-7.63	NER			
RAD51	-7.79	DSBs repair			
XRCC2	-11.47	DSBs repair			
**Decrease 2 - 5x**					
**Gene**	**Expression**	**Pathway**			
ABL1	-2.33	MMR			
APEX1	-3.48	BER			
ATM	-2.20	ATM/ATR pathway			
ATR	-2.04	ATM/ATR pathway			

ATRIP	-2.42	ATM/ATR pathway
ATRX	-4.86	DNA repair
DDB1	-2.97	NER
DDB2	-2.11	NER
FANCA	-2.81	DNA repair
LIG1	-2.06	BER
MBD4	-2.11	BER
MLH3	-2.14	MMR
MRE11A	-2.52	DSBs repair
MSH2	-3.69	MMR
NBN	-2.20	DSBs repair
NTHL1	-2.56	NER
PARP1	-3.41	BER
PMS1	-2.96	MMR
PPM1D	-4.60	ATM/ATR pathway
PRKDC	-3.27	DSBs repair
RAD17	-3.30	ATM/ATR pathway
RAD18	-2.11	DNA repair
RAD21	-2.99	DNA repair
RBBP8	-2.02	DNA repair
SIRT1	-3.54	NER
SMC1A	-3.78	ATM/ATR pathway
SUMO1	-2.22	DNA repair
TOPBP1	-2.45	ATM/ATR pathway
UNG	-2.95	BER
XRCC1	-2.08	BER
XRCC6	-3.18	DSBs repair
